# Utilising a Clinical Metabolomics LC-MS Study to Determine the Integrity of Biological Samples for Statistical Modelling after Long Term −80 °C Storage: A TOFI_Asia Sub-Study

**DOI:** 10.3390/metabo14060313

**Published:** 2024-05-29

**Authors:** Aidan Joblin-Mills, Zhanxuan E. Wu, Ivana R. Sequeira-Bisson, Jennifer L. Miles-Chan, Sally D. Poppitt, Karl Fraser

**Affiliations:** 1 Food Chemistry & Structure Team, AgResearch, Palmerston North 4410, New Zealand; magicboat27@outlook.com (Z.E.W.); karl.fraser@agresearch.co.nz (K.F.); 2 High-Value Nutrition National Science Challenge, Auckland 1145, New Zealand; i.sequeira@auckland.ac.nz (I.R.S.-B.); j.miles-chan@auckland.ac.nz (J.L.M.-C.); s.poppitt@auckland.ac.nz (S.D.P.); 3 School of Food and Nutrition, Massey University, Palmerston North 4410, New Zealand; 4 Human Nutrition Unit, School of Biological Sciences, University of Auckland, Auckland 1024, New Zealand; 5 Department of Medicine, University of Auckland, Auckland 1145, New Zealand

**Keywords:** frozen, −80 °C storage, lipidomics, metabolomics, multivariate modelling, predictions, ethnicity, comparison, confusion matrices

## Abstract

Biological samples of lipids and metabolites degrade after extensive years in −80 °C storage. We aimed to determine if associated multivariate models are also impacted. Prior TOFI_Asia metabolomics studies from our laboratory established multivariate models of metabolic risks associated with ethnic diversity. Therefore, to compare multivariate modelling degradation after years of −80 °C storage, we selected a subset of aged (≥5-years) plasma samples from the TOFI_Asia study to re-analyze via untargeted LC-MS metabolomics. Samples from European Caucasian (*n* = 28) and Asian Chinese (*n* = 28) participants were evaluated for ethnic discrimination by partial least squares discriminative analysis (PLS–DA) of lipids and polar metabolites. Both showed a strong discernment between participants ethnicity by features, before (Initial) and after (Aged) 5-years of −80 °C storage. With receiver operator characteristic curves, sparse PLS–DA derived confusion matrix and prediction error rates, a considerable reduction in model integrity was apparent with the Aged polar metabolite model relative to Initial modelling. Ethnicity modelling with lipids maintained predictive integrity in Aged plasma samples, while equivalent polar metabolite models reduced in integrity. Our results indicate that researchers re-evaluating samples for multivariate modelling should consider time at −80 °C when producing predictive metrics from polar metabolites, more so than lipids.

## 1. Introduction

Metabolomics analyses provide a comprehensive profiling of lipids and polar metabolites in biological samples, allowing researchers to identify changes in metabolic pathways and identify biomarkers associated with conditions such as metabolic syndrome and type 2 diabetes (T2D) [1,2,3].

In 2021, approximately 537 million people worldwide were reported as having diabetes. Furthermore, 118 million of these cases were Chinese adults aged 20–79 years, making China the country with the highest number of adults with diabetes in the world [4]. The prevalence of diabetes in China has increased rapidly over the past few decades, driven by a combination of factors, including changes in lifestyle, an aging population, and increased urbanization [5,6]. In particular, the prevalence of T2D amongst the Chinese population has been linked with the “Thin on the Outside Fat on the Inside” (TOFI) phenotype, where lean individuals present with visceral (VAT) and ectopic adipose tissue deposited in and around key organs such as the liver and pancreas [7,8,9]. Researchers have shown body mass index (BMI) to be a poor predictor of VAT and organ fat deposition and, in turn, a weaker indicator of T2D development within susceptible individuals [9,10,11]. Rather, researchers have established that the ratio of VAT to subcutaneous adipose tissue (VAT/SAT) provides for a more accurate predictor of an individual’s T2D susceptibility [12,13]. Previously published TOFI_Asia studies from our laboratory have monitored VAT/SAT ratios to further discern biological disparities between Asian Chinese and European Caucasian cohorts, modelling fasting plasma glucose (FPG) and associated metabolic markers, plus lipids and polar metabolite features for prediction of T2D risk [11,14,15]. This statistical and prediction modelling provides researchers with a non-invasive means to characterize complex conditions, such as the TOFI phenotype via easily accessible sample types.

Due to the complex nature of metabolomics analyses, including lack of standardization in sample collection, extraction protocols, instrument dependent parameters, and bioinformatics processing, inconsistencies and difficulties can arise when comparing results across studies, or evaluating prediction model outcomes in follow-up studies [16,17,18]. Metabolomics studies can be largely impacted by the number of detectable features, as certain structures may be missed across studies due to instrumental or environmental factors [19,20]. Metabolomics samples stored in freezers inevitably degrade over time due to temperature fluctuations, freeze–thaw cycles, and natural degradation of at-risk biomolecules [21,22]. Constituent proteins and nucleic acids such as DNA and RNA can degrade over time due to residual enzymatic activity and exposure to light, while lipids and polar metabolites can undergo oxidative degradation with exposure to the surrounding atmosphere [23,24]. Additionally, biological samples stored in freezers may be subject to contamination by microorganisms or other foreign materials, leading to changes in sample composition and compromised downstream analyses. As such, the rate and degree of sample degradation can depend on a multitude of factors, the type of sample, the storage conditions, and the length of time in storage [18,21,22].

Multivariate modelling is essential for dealing with large information rich datasets coming from clinical omics studies. Techniques such as principal component analysis (PCA) can identify and illustrate key sources of variation and clustered patterns among cohorts [25,26], while partial least squares discriminative analysis (PLS–DA) can formulate a weighted prediction towards a discrimination model within a cohort, highlighting the variables of most importance in establishing a classification [27]. While the strength of a PLS–DA model can be evaluated by goodness of fit (R2) and goodness of prediction (Q2) values, the validity of a model’s performance and its ability to predict a desired classification requires further due diligence [28]. For instance, receiver operating characteristic (ROC) curves can provide clinicians and data scientists with a useful means for evaluating the performance of such classification model’s, illustrating the trade-off between true positive and false positive rates across a range of thresholds [29,30]. The validity of a models performance can be visualized with a ROC curve and its calculated area under the curve (AUC) can quantify it [31].

Given the limited life span of liquid chromatography and mass spectrometry components, its common that a laboratories instrumentation and protocols change over years. In 2014, the metabo-RING initiative addressed concerns for possible discrepancies when implementing different metabolomics platforms for the same model. They concluded that different machines and methods for metabolomics are sufficiently comparative when modeling from the same set of samples [32]. While this study provided confidence in comparing metabolomics profiles across our different platforms, our concerns with the possible effects of long term −80 °C storage on metabolomics models remained.

We aimed to statistically project lipid and polar metabolite LCMS features before (Initial) and after (Aged) 5-year −80 °C storage to our previously published TOFI_Asia ethnicity (Caucasian vs. Chinese) model [15], to compare the preservation of predictive integrity among plasma samples. The comparison of multivariate models was achieved with PCA and PLS–DA modelling procedures, and further evaluation of the multivariate models was achieved by comparing the ROC curve and prediction error rates of each given model.

## 2. Materials and Methods

### 2.1. Study Cohort

The previously published TOFI_Asia studies were conducted at the Human Nutrition Unit, University of Auckland, New Zealand. All participants self-reported both parents of the same ethnic descent (i.e., European Caucasian or Asian Chinese) according to ethnic group profiles established by Statistics New Zealand [33]. Participants were recruited for both genders across a wide range of ages (20–70 years) and BMI (20–45 kg/m^2^) and were either normo-glycemic or had prediabetes based on the American Diabetes Association (ADA) criteria [34]. Exclusion criteria included significant weight gain or loss (>10%) in the previous 3 months, prior bariatric surgery, pregnancy, breastfeeding, current glucose-related medications (e.g., glucocorticoids) and significant current/prior history of disease including T2D.

A detailed description of the TOFI_Asia study cohort and relevant protocols can be found elsewhere [11,14,15]. From the total cohort, we randomly selected 28 European Caucasian and 28 Asian Chinese participants of equal distribution in gender (14 males and 14 females within each ethnic group) for the current sub-study. All sub-study participants had anthropometry and clinical biomarker data available and were categorized as normo-glycemic (*n* = 33) or with prediabetes (*n* = 23) based on FPG.

### 2.2. Metabolomic Analysis

#### 2.2.1. Chemicals and Reagents

Chemical solvents used for sample preparation and LC-MS analysis (methanol, isopropanol, formic acid, chloroform and acetonitrile) were purchased from Thermo Fisher Scientific (Auckland, New Zealand). Ammonium formate (Fluka™, HPLC grade) was obtained from Sigma-Aldrich (Auckland, New Zealand). Milli-Q^®^ ultrapure water was purchased from Merck Millipore (Bedford, MA, USA). All chemicals were of LC–MS grade except chloroform, which was of analytical grade.

#### 2.2.2. Sample Preparation

Both lipids and polar metabolites were extracted from our Initial TOFI_Asia plasma samples simultaneously using the Bligh and Dyer biphasic method [35]. Briefly, 100 µL plasma was mixed with 800 µL pre-chilled (−20 °C) CHCl_3_:MeOH (1:1, *v*/*v*), agitated for 30 s and stored at −20 °C for 60 min to allow protein precipitation. An additional 400 µL H_2_O was added to the tube, followed by 30 s of vortex-mixing and 10 min of 11,000 rpm centrifugation at 4 °C (Eppendorf Centrifuge 5427 R, Eppendorf, Hamburg, Germany). Subsequently, 200 µL of the upper aqueous layer and 200 µL of the lower organic layer were transferred into two separate 2 mL microcentrifuge tubes to isolate the extracted polar metabolites and lipids, respectively. Resulting extracts were dried down under a nitrogen stream and stored at −80 °C. On the day of instrumental analysis, dried polar extracts were reconstituted in 200 µL ACN:H_2_O (1:1, *v*/*v*) and lipid extracts in modified Folch solution (CHCl_3_:MeOH:H_2_O, 66:33:1, *v*/*v*/*v*).

A monophasic protocol for lipid and polar metabolite extractions was implemented with the Aged TOFI_Asia plasma samples, as previously outlined [36,37]. For the extraction of lipids, plasma samples were pre-thawed at 4 °C, agitated for 30 s and 10 µL was mixed with 100 µL of pre-chilled (−20 °C) BuOH:MeOH (1:1, *v*/*v*). Extraction samples were sonicated for 5 min and centrifuged at 11,000 rpm for 10 min at 4 °C. The supernatants were transferred to HPLC vials and stored at −80 °C. For the extraction of polar metabolites, 50 μL of the pre-thawed plasma was mixed with 450 μL pre-chilled (4 °C) ACN:H_2_O (9:1, *v*/*v*). Extraction tubes were sonicated for 5 min and centrifuged at 11,000 rpm for 10 min at 4 °C. Resulting supernatants were transferred to HPLC vials and stored at −80 °C.

All plasma samples were randomized into sectioned batches to run across consecutive days to account for batch-to-batch variation; pooled quality control (QC) samples were prepared by pooling aliquots from extracted samples, at the two time points, into a 15 mL falcon tube to mix, and subsequently distributed into a series of HPLC vials for intermittent QC injections after every ten samples across each study’s run order. A series of blank samples were prepared by substituting plasma for H_2_O within an extraction tube, which was subject to each respective extraction procedure, for incorporation into their relative run at the time point.

#### 2.2.3. Instruments and Conditions

External mass calibrations of the LCMS instruments prior to sample analyses were performed by flow injection of the calibration mix solution according to respective manufacturer’s instructions.

Initial TOFI_Asia lipids were analyzed with an Accela 1250 quaternary UHPLC pump coupled to a Q-Exactive hybrid quadrupole-Orbitrap (LTQ–Orbitrap) mass spectrometer (Thermo Fisher Scientific, Waltham, MA, USA). Chromatographic separation was carried out on a Acquity CSH™ C18 1.7 µm, 2.1 mm × 100 mm column (Waters, Milford, MA, USA) using the following solvent system: A = acetonitrile/H_2_O (6:4) with 10 mM ammonium formate and 0.1% formic acid, and B = isopropanol/acetonitrile (9:1) with 10 mM ammonium formate and 0.1% formic acid. An injection volume of 2 µL reconstituted sample was used and a mobile phase flow rate of 600 µL/min was implemented. A summary of the HPLC gradient program used is outlined in the table below (Table 1: Gradient programs). High resolution data (resolution 70,000) was acquired by full scan from *m*/*z* 200–2000 with source voltage of 3500 V electrospray ionization positive mode (ESI+) or −3600 V ESI negative mode (ESI−), capillary temperature of 275 °C, and sheath, auxiliary and sweep gas flow rates of 40, 10 and 5 arbitrary units, respectively. Data-dependent MS2 data were collected with a mass resolution set to 35,000 recording a mass range of *m*/*z* 200–2000 and maximum trap fill time of 250 ms (full scan mode) or 120 ms (MS2 scan mode). The isolation window of selected MS1 scans was ± 1.5 *m*/*z* with a normalized collision energy of 30 units.

Initial TOFI_Asia polar metabolites were analyzed with an Accela 1250 quaternary UHPLC pump coupled to an Exactive Orbitrap mass spectrometry (Thermo Fisher Scientific, Waltham, MA, USA). Chromatographic separation was carried out at 25 °C on a SeQuant^®^ ZIC^®^-pHILIC 5 µm 2.1 mm × 100 mm column (Merck, Darmstadt, Germany) using the following solvent system: A = H_2_O with 10 mM ammonium formate, B = ACN with 0.1% formic acid. An injection volume of 2 µL reconstituted sample was used and a mobile phase flow rate of 250 µL/min was implemented. A summary of the HPLC gradient program used is outlined in the Supplementary Materials (Supplementary Table S1). High resolution data (resolution 25,000) were acquired by full scan from *m*/*z* 55 to 1100 with source voltage of 4.0 kV for ESI+ and −4.0 kV for ESI−, capillary temperature of 325 °C, and sheath, auxiliary, and sweep gas flow rates of 40, 10, and five arbitrary units, respectively.

Aged TOFI_Asia lipids were analyzed with a Shimadzu Nexera-x2 UHPLC system coupled to Shimadzu LCMS-9030 quadrupole time-of-flight (Q–TOF) mass spectrometer (Shimadzu Scientific Instruments, Columbia, MD, USA). Chromatographic separation was carried out on a Acquity CSH™ C18 1.7 µm, 2.1 mm × 100 mm column (Waters, Milford, MA, USA) using the following solvent system: A = H_2_O/acetonitrile/isopropanol (5:3:2) with 10 mM ammonium formate, and B = H_2_O/acetonitrile/isopropanol (1:9:90) with 10 mM ammonium formate. An injection volume of 4 µL of sample was used and a mobile phase flow rate of 400 µL/min was implemented. A summary of the HPLC gradient program used is outlined (Supplementary Table S1: Gradient programs). High resolution data (resolution 30,000) measured full MS1 spectra from 250 to 1250 *m*/*z* across the entire chromatogram and collecting independent acquisition (DIA) data in 20 *m*/*z* windows from 300 to 1100 *m*/*z*, with a 0.6 s cycle time and collision energy of 25 normalized collision energy units. The source voltage was 4.0 kV for ESI+ and −4.0 kV for ESI, with a nebulizing gas flow of 2.0 L/min, heater gas flow of 10 L/min, interface temperature of 300 °C, drying gas flow of 10 L/min, desolvation line temperature of 250 °C, and heater block temperature of 400 °C. All drying and collision gasses used were nitrogen.

Aged TOFI_Asia polar metabolites were analyzed with a Shimadzu Nexera-x2 UHPLC system coupled to Shimadzu LCMS-9030 Q–TOF mass spectrometer (Shimadzu Scientific Instruments, Columbia, MD, USA). Chromatographic separation was carried out on a Thermo Accucore HILIC 2.6 µm, 2.1 × 100 mm column (Thermo Fisher Scientific, USA) using the following solvent system: A = H_2_O with 10 mM ammonium formate B = acetonitrile with 0.1% formic acid. An injection volume of 4 µL of sample was used and a mobile phase flow rate of 400 µL/min was implemented. A summary of the HPLC gradient program used is outlined in a table below (Table 1: Gradient programs). High resolution data (resolution 30,000) measured full MS1 spectra from 70 to 1000 *m*/*z* across the entire chromatogram and collecting DIA data in 20 *m*/*z* windows from 70 to 900 *m*/*z*, with a 0.6 s cycle time and collision energy of 25 normalized collision energy units. The source voltage was 4.0 kV for ESI+ and −4.0 kV for ESI, with a nebulizing gas flow of 2.0 L/min, heater gas flow of 10 L/min, interface temperature of 300 °C, drying gas flow of 10 L/min, desolvation line temperature of 250 °C, and heater block temperature of 400 °C. All drying and collision gasses used were nitrogen.

#### 2.2.4. Metabolomics Data Processing

Initial samples’ lipid and polar metabolite raw datafiles were converted to mzXML format using ProteoWizard tool MSconvert (v 3.0.1818). Open mzXML data files were pre-processed for features by untargeted peak filtering, peak alignment, and peak filling parameters with the XCMS package (v3.0.2) in the R programming environment (v3.2.2) [38]. Features not detected in 100% of the QC samples were excluded from the analysis and resulting extracted ion chromatograms were manually examined to filter poorly integrated peaks generated by the diffreport function. Signal drift and batch effects were corrected for by LOESS algorithm in the online W4M Galaxy environment, and feature filtering with a <30% coefficient of variation limit among QC samples was applied [39].

Aged samples lipid and polar metabolite raw datafiles were converted to centroid mzML format using the Shimadzu file converter and then explored using the open access software package MS–DIAL [40]. MS–DIAL was used to perform peak detection, retention time alignment, grouping, gap filling, and run order and batch correction using the LOW–ESS algorithm. MS–DIAL was also used to search and annotate the aligned peaks found in the DIA MS/MS spectral data collected. The lipidomics results were searched against the built-in lipid library containing 257,000 in silico generated MS/MS lipid fragmentation spectra, and Metabolomics results were searched against the open access FeihnLib libraries and an in-house library of putative isotope ratio outlier analysis (IROA) standards [41,42]. QC samples were used for a loess adjustment of any run-order effects or loss in instrumental performance. The data matrix was exported for further filtering of unreliably measured peaks relative to QC samples via relative standard deviation (RSD > 0.3) and the resultant data matrix was used for downstream statistical analyses.

### 2.3. Statistical Analysis

To determine if the quality of a statistical model is preserved among biological samples after long term −80 °C storage, we chose to replicate the ethnicity (European Caucasian vs. Asian Chinese) modeling presented in prior TOFI_Asia metabolomics studies [14,15]. All lipid and polar metabolite data matrices were subject to k-nearest neighbors imputation for missing data points and were log transformed, summary scaled and mean centered to normalize the matrix in preparation for multivariate statistics [43].

PCA and PLS–DA were performed in SIMCA software version 16 (Sartorius, Umea, Sweden). All cross validations and sparse PLS–DA (sPLS–DA) procedures were performed within the Multivariate INTegrative (MINT) mixOmics package in R programming environment (v3.2.2) [28,44]. Each sPLS–DA implemented a least absolute selection and shrinkage operator (LASSO) penalization when tuning the variable selection parameters. Each resulting sPLS–DA model’s predictive performance was evaluated with ROC curves, as briefly outlined [31]. By setting a range of thresholds to evaluate against the sPLSDA tuned Ethnicity models, false positive rates ($FPR=\frac{FN}{FP+TN}$) and true positive rates ($TPR=\frac{TP}{TP+FN}$) were calculated and plotted across the y and *x*-axis, respectively, forming a ROC graph for each Initial and Aged lipid and polar metabolite dataset for the prediction of ethnicity, and an AUC value was calculated for each ROC curve [29]. By using the pre-tuned sparse parameters to store a prediction score against a single component for each model, a confusion matrix was established and prediction error scores were calculated, providing a quantifiable comparison of each sPLS–DA prediction model’s performance [45].

## 3. Results

### 3.1. General Trends

Equal parts male (*n* = 14) to female (*n* = 14), Caucasian (*n* = 14) and Chinese (*n* = 14) TOFI_Asia study participants, aged 18–68 years, with a BMI range between 21–37 kg/m^2^, had TOFI_Asia LCMS metabolomics data retrieved (Initial), and their respective –80 °C plasma samples thawed and re-analysed via current LCMS protocols (Aged) for comparative modelling. A total of 188 lipids and 78 polar metabolite features were identified from all Initial and Aged plasma samples when comparing each LCMS feature by their mass charge value (equal to 2 decimal points) and their relative retention time value (Supplementary Tables S1 and S2).

**Figure 1:**
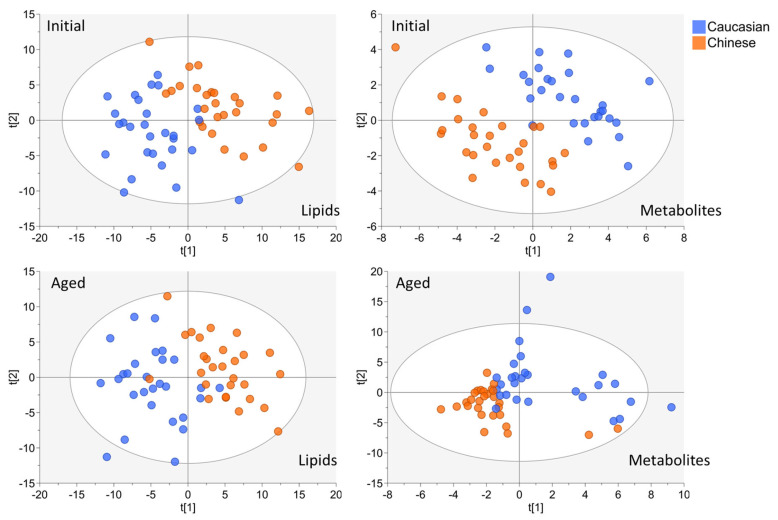
Partial least squares discriminative analysis of both Initial and Aged lipid and metabolite datasets for Ethnicity discrimination (Caucasian vs. Chinese). For each model, participant symbols were colored according to ethnicity (Caucasian blue symbols, Chinese orange symbols).

Implementation of PCA on both lipid and polar metabolite features showed a weak but detectable visual separation between Caucasian (green symbols) and Chinese (blue symbols) participants when projecting each Initial and Aged dataset to multiple components (Supplementary Figure S1). Visual separation by ethnicity was significantly improved with PLS–DA of all lipid and metabolite models (Figure 1). These results provided confidence in ethnicity as a valid choice for evaluating the degradation or preservation of multivariate models among the TOFI_Asia studies outcomes. The Aged lipid and Aged polar metabolite PCA models presented higher cumulative R2X values relative to their respective Initial-omic model values, suggesting a moderate increase in global variation among the cohort’s lipid and polar metabolites when the samples were thawed from −80 °C after five years (Table 1).

### 3.2. The Maintenance of Initial vs. Aged Discriminative Potential of TOFI_Asia Ethnicity Models

**Figure 2:**
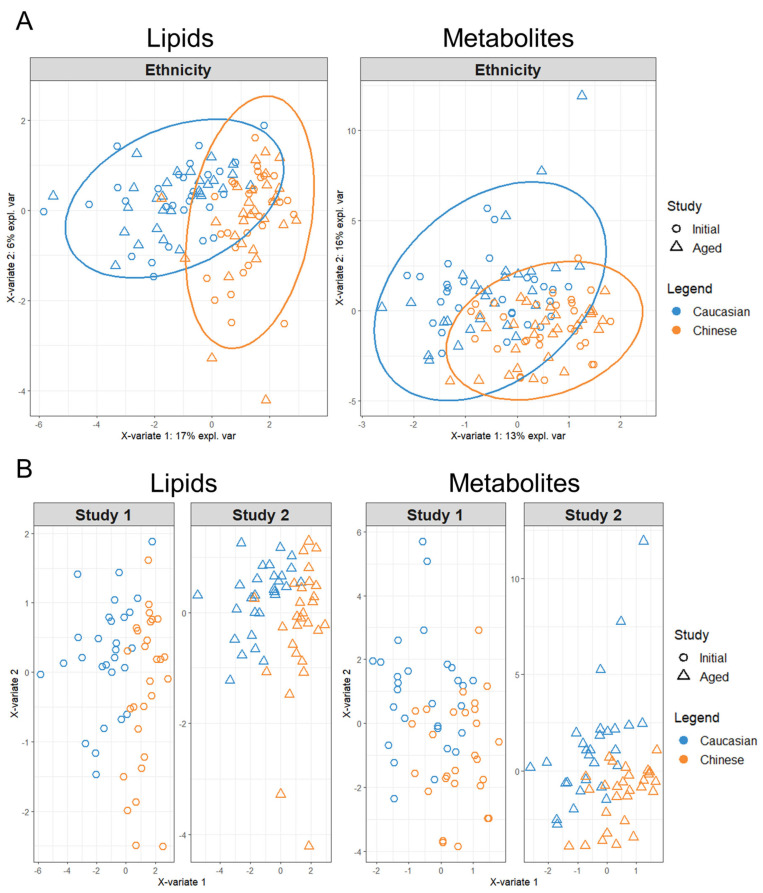
Sparse tuning of block PLS–DA lipid and polar metabolite models for the discrimination of Ethnicity via the MixOmics MINT analysis. For each model, participant symbols were colored according to ethnicity (Caucasian blue symbols, Chinese orange symbols). (**A**) Sparse tuned block PLS–DA plots of respective lipid and metabolite Ethnicity models, calculated from the combination of Initial and Age study features as collective discrimination analysis. (**B**) Sparse tuned block PLS–DA plots of respective lipid and metabolite Ethnicity models as separated by Initial (Study 1) and Aged (Study 2) panels for comparison of discrimination analysis.

PLS–DA calculated cumulative R2Y values decreased in both Aged lipid and Aged polar metabolite ethnic discrimination models, relative to their Initial model projections (Table 1). This indicated a minor decrease in strength for predicting the European Caucasian vs. Asian Chinese model among the Aged samples features. Regardless, each model’s performance value and score plot indicates strong separation of participants by ethnicity (Figure 1). By implementing the MixOmics block- sPLS–DA function (MINT), we tuned and evaluated the Initial and Aged plasma samples collectively to further evaluate the discrimination of the TOFI_sub-cohort by ethnicity (Figure 2A).

By using the LASSO penalty, MINT analysis of the combined lipidomics dataset reduced the number of variables required to classify ethnicity down to eight after 10-fold cross validation and parameter tuning. The refined sPLS–DA model revealed that the first component (x-variate 1) could explain considerably more co-variance by 17% across the *x*-axis than the second component (x-variate 2) with only 6% along the *y*-axis. In contrast, both components for the combined polar metabolites model explained relatively more co-variance between the first and second component with 13% and 16% explained covariance, respectively. The polar metabolites MINT model was a result of 40 LASSO selected variables classifying ethnicity, indicating more global variation among participants’ metabolites other than those contributing to the ethnicity model, irrespective of Initial or Aged plasma sampling.

The degree of disparity among the lipid and polar metabolites’ datasets was further evaluated by splitting the MINT analysis into their respective Initial and Aged study panels (Figure 2B). Splitting the sPLS–DA MINT lipidomics datasets into their respective Initial and Aged analyses showed a clustering of participants within the Aged dataset with a weak reduction in co-variance along X-variate 2. This clustering of participants with Aged plasma sampling was apparent within the polar metabolite’s panels, but the Aged metabolites’ dataset presented a strong increase in variation across both X-variate axes, relative to their Initial participants’ projections.

### 3.3. Evaluation of Initial and Aged TOFI_Asia Ethnicity Prediction Models

**Figure 3:**
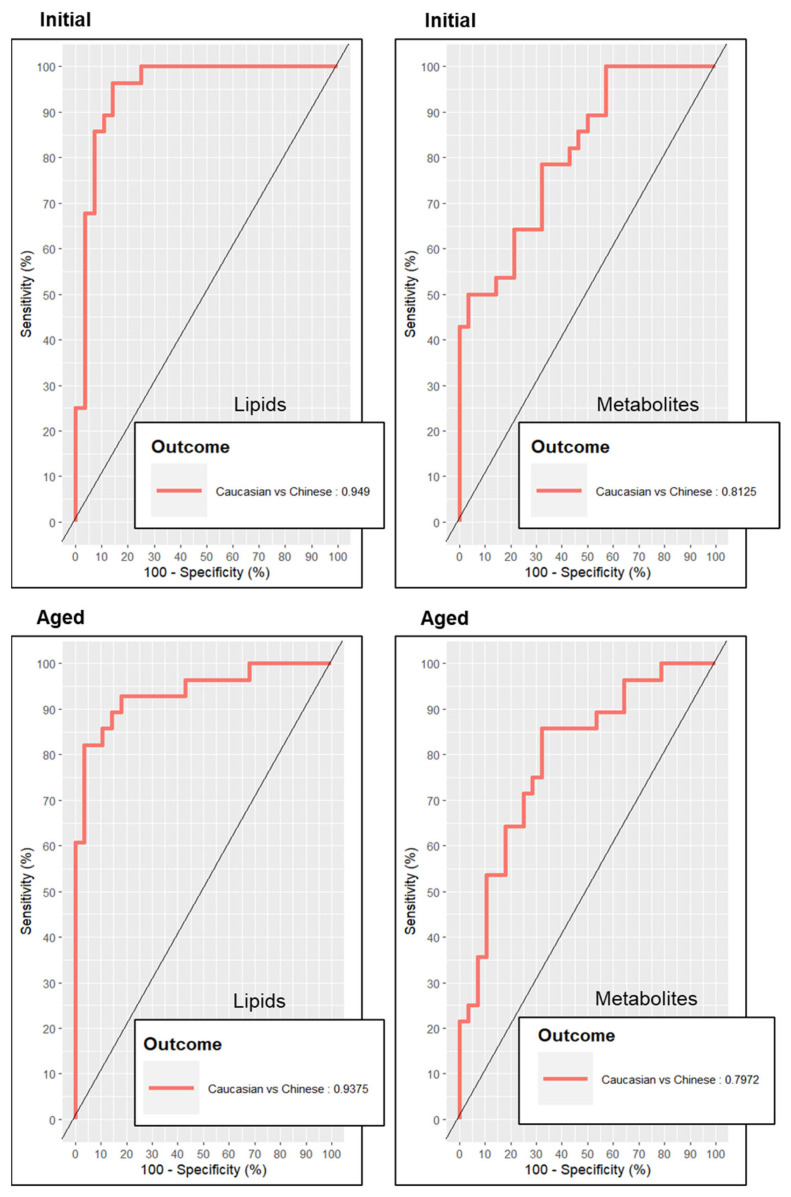
Receiver operator curves depicting the predictive accuracy of each metabolomics model for discrimination of ethnicity. The predictive accuracy of receiver operator curves presented as “outcomes” depicting area under the curve (AUC) values.

After penalty tuning and establishing each Initial and Aged sPLS–DA model for ethnic discrimination, we chose to evaluate the predictive quality of each trained model with ROC curves (Figure 3). ROC curves and their calculated AUC values indicated a greater performance in predicting the cohort’s ethnic discrimination from both Initial lipid and Initial polar metabolite models relative to their respective Aged models. Additionally, ROC analysis determined that both Initial and Aged lipidomic models were stronger prediction models for ethnic discrimination than their polar metabolite models.

Further evaluation of each model’s potential was achieved by calculating a confusion matrix with modelling error rate scores (Table 2A,B). Both the confusion matrices and subsequent error rates supported a significant decline in the prediction power of polar metabolite markers for discrimination of ethnicity after prolonged −80 °C storage. Surprisingly, the Aged lipidomics profile resulted in a weak increase in prediction, indicating that regardless, lipids are more stable features for prediction modelling, in particular the discrimination of ethnicity among the TOFI_Asia study sub-cohort, relative to polar metabolites.

## 4. Discussion

To the best of our knowledge, this is the first study examining the integrity of multivariate models from plasma samples stored at −80 °C for several years, rather than the stability of the individual lipids or polar metabolites used to establish the multivariate models. Our study re-evaluated the TOFI_Asia ethnicity models projected from plasma sample lipids and polar metabolites, as previously published [14,15]. A sub-cohort of an equal number of Asian Chinese and European Caucasian TOFI_Asia participants was selected at random from the original cohort, and their plasma samples thawed and re-analysed via our updated TOF mass spectrometry protocols. Lipids and polar metabolites were subjected to multivariate modelling to determine their homogeneous/heterogenous changes, and more importantly, their relative characterization of the TOFI_Asia ethnicity models. In turn, we compared the strength of ethnicity discrimination established from the same plasma samples before and after 5 years of −80 °C storage.

Interestingly, with respect to our calculated PCA, the R2X and Q2 values for both Aged plasma lipid and polar metabolite models indicated an increase in variance relative to respective Initial plasma models. When colored according to participants’ “Ethnic” denotation within the metadata, our PCA plots indicated a weak but visible separation of participants’ ethnicity among both Initial and Aged lipid and polar metabolite features (Supplementary Figure S1). This degree of unsupervised ethnicity discrimination resulted in a strong ethnicity discernment by PLS–DA (Supplementary Figure S2 and Table S1). Although both lipid and polar metabolite Aged plasma models R2Y values indicated a minor loss in model power when compared to their Initial plasma samples, all models provided moderate to high predictive power (Table 1), in turn providing bioinformaticians with a degree of confidence in −80 °C storage practices.

By implementing a sparse tuning process within the block PLS–DA procedure, we found that the combined polar metabolites model required significantly more features to classify ethnic cohorts, relative to the number required from lipids as modelling features (i.e., 40 vs. 8, respectively). This reflects the overall strength of stability among lipid features relative to polar metabolites when establishing the TOFI_Asia ethnicity models after prolonged cryogenic conditions. ROC and confusion matrices supported the notion that lipids could discriminate ethnicity more so than polar metabolites, but also showed a reduction in modelling stability from Aged plasma samples. While ROC–AUC values indicated a minor loss in model stability between Initial and Aged plasma lipid models, confusion error rates suggested a minor increase in predictive integrity with the Aged plasma lipid model. In contrast, both ROC–AUC values and confusion error rates indicated a significant collapse in model stability and predictive power with the Aged polar metabolites model relative to the Initial polar metabolites model, and both Initial and Aged lipid models. This suggests a detrimental loss of predictive power from polar metabolite features after additional years of −80 °C storage.

The structural and annotative knowledge of small molecules and lipids has become increasingly comprehensive over the years. The Human Metabolome Database (HMDB) reported a total of 114,100 metabolites, detected and predicted, all with taxonomic and ontological mappings [46]. LIPIDMAPS Consortium estimated a conservative number of discrete lipid structures in the order of 200,000, given the many variations in acyl/alkyl chains and structural permutations [47]. The collective efforts of geneticists and clinicians have characterized a significant portion of the human metabolome, reporting 41,993 small-molecule metabolites [48,49]. Untargeted clinical studies generally report anywhere from 100–200 metabolites within batched LCMS runs of human plasma [14,50]. Previous attempts at covering the human plasma lipidome report approximately 600 lipid species, comprising six main mammalian lipid categories [51]. Several studies have evaluated the degradation potential of metabolites, lipid species and lipid classes with respect to freezing, thawing, storage conditions, time on a bench or time within an incubator [52,53,54]. Regardless of the inevitable degradation of commonly reported features in clinical settings, we hypothesized that the discriminant degradation of such metabolites and lipids would have a minor impact on our chosen multivariate models, with the outcome being a change in predictive strength, more than a complete loss of predictive capability.

Although our study compared features derived from different methods of extraction (bi-vs. monophasic extraction) and means of acquisition (Orbitrap vs. TOF mass spectrometry), previous reports [55,56,57] along with our in-house optimizing [58] strongly indicated that a change from biphasic to monophasic extraction protocols for lipids and polar metabolites would yield comparable features for the current study outcomes. Furthermore, the metabo-RING study, which evaluated the reliability of untargeted metabolomics data across various laboratories with their respective LCMS instruments and in-house extraction protocols, reported comparable results from the same urine and plasma samples [32]. In particular, the groups comparing Q–TOF and LTQ–Orbitrap performances reported no effect due to instrumentation. For more direct comparison of un-annotated LCMS spectra, a group has developed an iterative alignment algorithm to account for retention time drifts between batches when processing features for selection [59]. Unfortunately, due to the algorithm’s limitations with aligning spectra with severe retention time shifts (and in our case, two different manufacturers chromatography columns and slightly different solvent gradients), our features resulting from two methodologies of LC-MS acquisition were out of scope for current tools alignment capabilities, so required manual curation for final selection, posing the greatest limitation to direct comparison of the data.

## 5. Conclusions

In conclusion, the comparison of lipid and polar metabolite features from plasma samples, after varying lengths of −80 °C storage (i.e., post collection vs. 5 years), indicated that the predictive quality of the polar metabolite features were impacted by cryogenic “Ageing”. The prediction of the previously established TOFI_Asia ethnicity model showed only a weak to negligible reduction in PLS–DA strength when modelling ethnicity by both lipid and metabolite features. However, more thorough evaluation of the lipid and metabolite models by sparse-PLS–DA and subsequent ROC analysis revealed a greater detriment in metabolite modelling after 5 years at −80 °C storage, while lipid modelling indicated greater stability for modelling potential. Although the effect of long term −80 °C storage did have an impact, both sets of lipid and metabolite features maintained valid strength to accurately predict the pre-established TOFI_Asia ethnicity models.

## Figures and Tables

**Table 1:** Comparison of Initial vs. Aged lipid and metabolomics multivariate models.

Analysis			Initial			Aged		
Samples	Number of Variables	Models	R2X(cum)	R2Y(cum)	Q2(cum)	R2X(cum)	R2Y(cum)	Q2(cum)
Lipids	188	PCA	0.75		0.56	0.82		0.67
		PLS–DA	0.41	0.65	0.47	0.4	0.64	0.49
Metabolites	84	PCA	0.57		0.12	0.73		0.56
		PLS–DA	0.22	0.8	0.57	0.65	0.76	0.54

Principle component analysis (PCA) and partial least squares discriminative analysis (PLS–DA) comparing both the latent modelling potential of lipids and polar metabolites from Initial and Aged plasma samples for feature hetero/homogeneity and ethnic discrimination, respectively.

**Table 2:** Confusion matrix and prediction error rates for determining the accuracy of sPLS–DA ethnic discrimination models for Initial and Aged lipidomic and metabolomic datasets.

(**A**): Ethnicity model confusion matrix.				
**Lipids**			**Metabolites**	
**Initial**	**Predicted as Caucasian**	**Predicted as Chinese**	**Predicted as Caucasian**	**Predicted as Chinese**
Caucasian	23	5	19	9
Chinese	5	23	7	21
Aged	Predicted as Caucasian	Predicted as Chinese	Predicted as Caucasian	Predicted as Chinese
Caucasian	22	6	14	14
Chinese	3	25	8	20
(**B**): Ethnicity model confusion error rates.				
Confusion error rates				
	**Lipids**	**Metabolites**		
Initial	0.18	0.29		
Aged	0.16	0.39		

## Data Availability

The data presented in this study are available on request from the corresponding author. The data are not publicly available due to ethics restrictions.

## Supplementary Materials

The following supporting information can be downloaded at: https://www.mdpi.com/article/10.3390/metabo14060313/s1, Figure S1: PCA of Initial vs. Aged plasma lipid and polar metabolites colored by ethnicity (Caucasian blue symbols, Chinese orange symbols). Table S1: HPLC gradient elution programs ran for both Initial and Aged study samples for both lipidomics and HILIC metabolomics untargeted LC-MS methodologies. Table S2A: Comparative list of lipidomics features found in both Initial and Aged plasma samples, labelled by relative *m*/*z* and retention time found. Table S2B: List of polar metabolite features found in both Initial and Aged plasma samples, labelled by relative *m*/*z* and retention time found.

## References

[1] Jin Q., Ma R.C.W. (2021). Metabolomics in Diabetes and Diabetic Complications: Insights from Epidemiological Studies. Cells.

[2] Newgard C.B. (2017). Metabolomics and Metabolic Diseases: Where Do We Stand?. Cell Metab..

[3] Savolainen O., Fagerberg B., Vendelbo Lind M., Sandberg A.-S., Ross A.B., Bergström G. (2017). Biomarkers for predicting type 2 diabetes development—Can metabolomics improve on existing biomarkers?. PLoS ONE.

[4] Magliano D.J., Boyko E.J. (2021). IDF Diabetes Atlas.

[5] Hu C., Jia W. (2018). Diabetes in China: Epidemiology and Genetic Risk Factors and Their Clinical Utility in Personalized Medication. Diabetes.

[6] Shen X., Vaidya A., Wu S., Gao X. (2016). The Diabetes Epidemic in China: An Integrated Review of National Surveys. Endocr. Pract..

[7] Nazare J.A., Smith J.D., Borel A.L., Haffner S.M., Balkau B., Ross R., Massien C., Alméras N., Després J.P. (2012). Ethnic influences on the relations between abdominal subcutaneous and visceral adiposity, liver fat, and cardiometabolic risk profile: The International Study of Prediction of Intra-Abdominal Adiposity and Its Relationship With Cardiometabolic Risk/Intra-Abdominal Adiposity. Am. J. Clin. Nutr..

[8] Thomas E.L., Parkinson J.R., Frost G.S., Goldstone A.P., Doré C.J., Mccarthy J.P., Collins A.L., Fitzpatrick J.A., Durighel G., Taylor-Robinson S.D. (2012). The Missing Risk: MRI and MRS Phenotyping of Abdominal Adiposity and Ectopic Fat. Obesity.

[9] Wulan S.N., Westerterp K.R., Plasqui G. (2010). Ethnic differences in body composition and the associated metabolic profile: A comparative study between Asians and Caucasians. Maturitas.

[10] Yoon K.-H., Lee J.-H., Kim J.-W., Cho J.H., Choi Y.-H., Ko S.-H., Zimmet P., Son H.-Y. (2006). Epidemic obesity and type 2 diabetes in Asia. Lancet.

[11] Sequeira I.R., Yip W., Lu L., Jiang Y., Murphy R., Plank L., Zhang S., Liu H., Chuang C.L., Vazhoor-Amarsingh G. (2020). Visceral Adiposity and Glucoregulatory Peptides are Associated with Susceptibility to Type 2 Diabetes: The TOFI_Asia Study. Obesity.

[12] Fox C.S., Massaro J.M., Hoffmann U., Pou K.M., Maurovich-Horvat P., Liu C.-Y., Vasan R.S., Murabito J.M., Meigs J.B., Cupples L.A. (2007). Abdominal Visceral and Subcutaneous Adipose Tissue Compartments. Circulation.

[13] Tang L., Zhang F., Tong N. (2016). The association of visceral adipose tissue and subcutaneous adipose tissue with metabolic risk factors in a large population of Chinese adults. Clin. Endocrinol..

[14] Wu Z.E., Fraser K., Kruger M.C., Sequeira I.R., Yip W., Lu L.W., Plank L.D., Murphy R., Cooper G.J.S., Martin J.-C. (2021). Untargeted metabolomics reveals plasma metabolites predictive of ectopic fat in pancreas and liver as assessed by magnetic resonance imaging: The TOFI_Asia study. Int. J. Obes..

[15] Wu Z.E., Fraser K., Kruger M.C., Sequeira I.R., Yip W., Lu L.W., Plank L.D., Murphy R., Cooper G.J.S., Martin J.-C. (2020). Metabolomic signatures for visceral adiposity and dysglycaemia in Asian Chinese and Caucasian European adults: The cross-sectional TOFI_Asia study. Nutr. Metab..

[16] Gertsman I., Barshop B.A. (2018). Promises and pitfalls of untargeted metabolomics. J. Inherit. Metab. Dis..

[17] Johnson C.H., Gonzalez F.J. (2012). Challenges and opportunities of metabolomics. J. Cell. Physiol..

[18] Ulmer C.Z., Koelmel J.P., Jones C.M., Garrett T.J., Aristizabal-Henao J.J., Vesper H.W., Bowden J.A. (2021). A Review of Efforts to Improve Lipid Stability during Sample Preparation and Standardization Efforts to Ensure Accuracy in the Reporting of Lipid Measurements. Lipids.

[19] Alseekh S., Aharoni A., Brotman Y., Contrepois K., D’Auria J., Ewald J., Ewald J.C., Fraser P.D., Giavalisco P., Hall R.D. (2021). Mass spectrometry-based metabolomics: A guide for annotation, quantification and best reporting practices. Nat. Methods.

[20] Spicer R.A., Salek R., Steinbeck C. (2017). A decade after the metabolomics standards initiative it’s time for a revision. Sci. Data.

[21] Wagner-Golbs A., Neuber S., Kamlage B., Christiansen N., Bethan B., Rennefahrt U., Schatz P., Lind L. (2019). Effects of Long-Term Storage at -80 °C on the Human Plasma Metabolome. Metabolites.

[22] Reis G.B., Rees J.C., Ivanova A.A., Kuklenyik Z., Drew N.M., Pirkle J.L., Barr J.R. (2021). Stability of lipids in plasma and serum: Effects of temperature-related storage conditions on the human lipidome. J. Mass Spectrom. Adv. Clin. Lab.

[23] Tateishi-Karimata H., Sugimoto N. (2014). Structure, stability and behaviour of nucleic acids in ionic liquids. Nucleic Acids Res..

[24] Lindahl T., Nyberg B. (1972). Rate of depurination of native deoxyribonucleic acid. Biochemistry.

[25] Vargason T., Howsmon D., Mcguinness D., Hahn J. (2017). On the Use of Multivariate Methods for Analysis of Data from Biological Networks. Processes.

[26] Papaioannou A., Simeonov V., Plageras P., Dovriki E., Spanos T. (2007). Multivariate statistical interpretation of laboratory clinical data. Open Med..

[27] Fonville J.M., Richards S.E., Barton R.H., Boulange C.L., Ebbels T.M.D., Nicholson J.K., Holmes E., Dumas M.-E. (2010). The evolution of partial least squares models and related chemometric approaches in metabonomics and metabolic phenotyping. J. Chemom..

[28] Rohart F., Eslami A., Matigian N., Bougeard S., Lê Cao K.-A. (2017). MINT: A multivariate integrative method to identify reproducible molecular signatures across independent experiments and platforms. BMC Bioinform..

[29] Hajian-Tilaki K. (2013). Receiver Operating Characteristic (ROC) Curve Analysis for Medical Diagnostic Test Evaluation. Casp. J. Intern. Med..

[30] Bünger R., Mallet R.T. (2016). Metabolomics and Receiver Operating Characteristic Analysis. Crit. Care Med..

[31] Greiner M., Pfeiffer D., Smith R.D. (2000). Principles and practical application of the receiver-operating characteristic analysis for diagnostic tests. Prev. Vet. Med..

[32] Martin J.-C., Maillot M., Mazerolles G., Verdu A., Lyan B., Migné C., Defoort C., Canlet C., Junot C., Guillou C. (2015). Can we trust untargeted metabolomics? Results of the metabo-ring initiative, a large-scale, multi-instrument inter-laboratory study. Metabolomics.

[33] Aotearoa S.N.T. Stats NZ (2019). 2018 Census Data User Guide. http://nzdotstat.stats.govt.nz/.

[34] American Diabetes Association (2021). 2. Classification and Diagnosis of Diabetes: Standards of Medical Care in Diabetes-2021. Diabetes Care.

[35] Bligh E.G., Dyer W.J. (1959). A rapid method of total lipid extraction and purification. Can. J. Biochem. Physiol..

[36] Lepoittevin M., Blancart-Remaury Q., Kerforne T., Pellerin L., Hauet T., Thuillier R. (2023). Comparison between 5 extractions methods in either plasma or serum to determine the optimal extraction and matrix combination for human metabolomics. Cell. Mol. Biol. Lett..

[37] Alshehry Z., Barlow C., Weir J., Zhou Y., Mcconville M., Meikle P. (2015). An Efficient Single Phase Method for the Extraction of Plasma Lipids. Metabolites.

[38] Smith C.A., Want E.J., O’Maille G., Abagyan R., Siuzdak G. (2006). XCMS: Processing mass spectrometry data for metabolite profiling using nonlinear peak alignment, matching, and identification. Anal. Chem..

[39] Giacomoni F., Le Corguille G., Monsoor M., Landi M., Pericard P., Petera M., Duperier C., Tremblay-Franco M., Martin J.-F., Jacob D. (2015). Workflow4Metabolomics: A collaborative research infrastructure for computational metabolomics. Bioinformatics.

[40] Tsugawa H., Cajka T., Kind T., Ma Y., Higgins B., Ikeda K., Kanazawa M., Vandergheynst J., Fiehn O., Arita M. (2015). MS-DIAL: Data-independent MS/MS deconvolution for comprehensive metabolome analysis. Nat. Methods.

[41] Kind T., Wohlgemuth G., Lee D.Y., Lu Y., Palazoglu M., Shahbaz S., Fiehn O. (2009). FiehnLib: Mass Spectral and Retention Index Libraries for Metabolomics Based on Quadrupole and Time-of-Flight Gas Chromatography/Mass Spectrometry. Anal. Chem..

[42] Beecher C., de Jong F. (2019). Using IROA-Based Internal Standard Normalization to Minimize Non-IROA Metabolite Variation.

[43] Worley B., Powers R. (2012). Multivariate Analysis in Metabolomics. Curr. Metabolomics.

[44] R Core Team (2021). R: A Language and Environment for Statisitical Computing.

[45] Rohart F., Gautier B., Singh A., Lê Cao K.-A. (2017). mixOmics: An R package for ‘omics feature selection and multiple data integration. PLoS Comput. Biol..

[46] Wishart D.S., Feunang Y.D., Marcu A., Guo A.C., Liang K., Vázquez-Fresno R., Sajed T., Johnson D., Li C., Karu N. (2018). HMDB 4.0: The human metabolome database for 2018. Nucleic Acids Res..

[47] Fahy E., Cotter D., Sud M., Subramaniam S. (2011). Lipid classification, structures and tools. Biochim. Biophys. Acta (BBA)—Mol. Cell Biol. Lipids.

[48] Qiu S., Cai Y., Yao H., Lin C., Xie Y., Tang S., Zhang A. (2023). Small molecule metabolites: Discovery of biomarkers and therapeutic targets. Signal Transduct. Target. Ther..

[49] Gallois A., Mefford J., Ko A., Vaysse A., Julienne H., Ala-Korpela M., Laakso M., Zaitlen N., Pajukanta P., Aschard H. (2019). A comprehensive study of metabolite genetics reveals strong pleiotropy and heterogeneity across time and context. Nat. Commun..

[50] Trabado S., Al-Salameh A., Croixmarie V., Masson P., Corruble E., Fève B., Colle R., Ripoll L., Walther B., Boursier-Neyret C. (2017). The human plasma-metabolome: Reference values in 800 French healthy volunteers; impact of cholesterol, gender and age. PLoS ONE.

[51] Quehenberger O., Dennis E.A. (2011). The Human Plasma Lipidome. N. Engl. J. Med..

[52] Torell F., Bennett K., Rännar S., Lundstedt-Enkel K., Lundstedt T., Trygg J. (2017). The effects of thawing on the plasma metabolome: Evaluating differences between thawed plasma and multi-organ samples. Metabolomics.

[53] Buchanan J.L., Tormes Vaquerano J., Taylor E.B. (2022). Isolated Effects of Plasma Freezing versus Thawing on Metabolite Stability. Metabolites.

[54] Shang X., Du J., Zhao Y., Tian J., Jiang S. (2021). Effect of Multiple Freeze-Thaw Cycles on Lipid Degradation and Lipid Oxidation of Grass Carp Surimi Containing Different Amounts of Pork Back Fat. Food Sci. Anim. Resour..

[55] Gil A., Zhang W., Wolters J.C., Permentier H., Boer T., Horvatovich P., Heiner-Fokkema M.R., Reijngoud D.-J., Bischoff R. (2018). One- vs two-phase extraction: Re-evaluation of sample preparation procedures for untargeted lipidomics in plasma samples. Anal. Bioanal. Chem..

[56] Mchugh C., Flott T., Schooff C., Smiley Z., Puskarich M., Myers D., Younger J., Jones A., Stringer K. (2018). Rapid, Reproducible, Quantifiable NMR Metabolomics: Methanol and Methanol: Chloroform Precipitation for Removal of Macromolecules in Serum and Whole Blood. Metabolites.

[57] Southam A.D., Haglington L.D., Najdekr L., Jankevics A., Weber R.J.M., Dunn W.B. (2020). Assessment of human plasma and urine sample preparation for reproducible and high-throughput UHPLC-MS clinical metabolic phenotyping. Analyst.

[58] Abshirini M., Cabrera D., Fraser K., Siriarchavatana P., Wolber F.M., Miller M.R., Tian H.S., Kruger M.C. (2021). Mass Spectrometry-Based Metabolomic and Lipidomic Analysis of the Effect of High Fat/High Sugar Diet and Greenshell^TM^ Mussel Feeding on Plasma of Ovariectomized Rats. Metabolites.

[59] Zhang Z. (2012). Retention Time Alignment of LC/MS Data by a Divide-and-Conquer Algorithm. J. Am. Soc. Mass Spectrom..

