# Supplementary material for: Utilising a Clinical Metabolomics LC-MS Study to Determine the Integrity of Biological Samples for Statistical Modelling after Long Term −80 °C Storage: A TOFI_Asia Sub-Study
Source: Metabolites. 2024 May 29;14(6):313. doi: 10.3390/metabo14060313 (PMC11205627; doi:10.3390/metabo14060313)
Supplement: Supplementary file 1 [file metabolites-14-00313-s001.zip › metabolites-2990754-supplementary.pdf]

Supplementary Figures

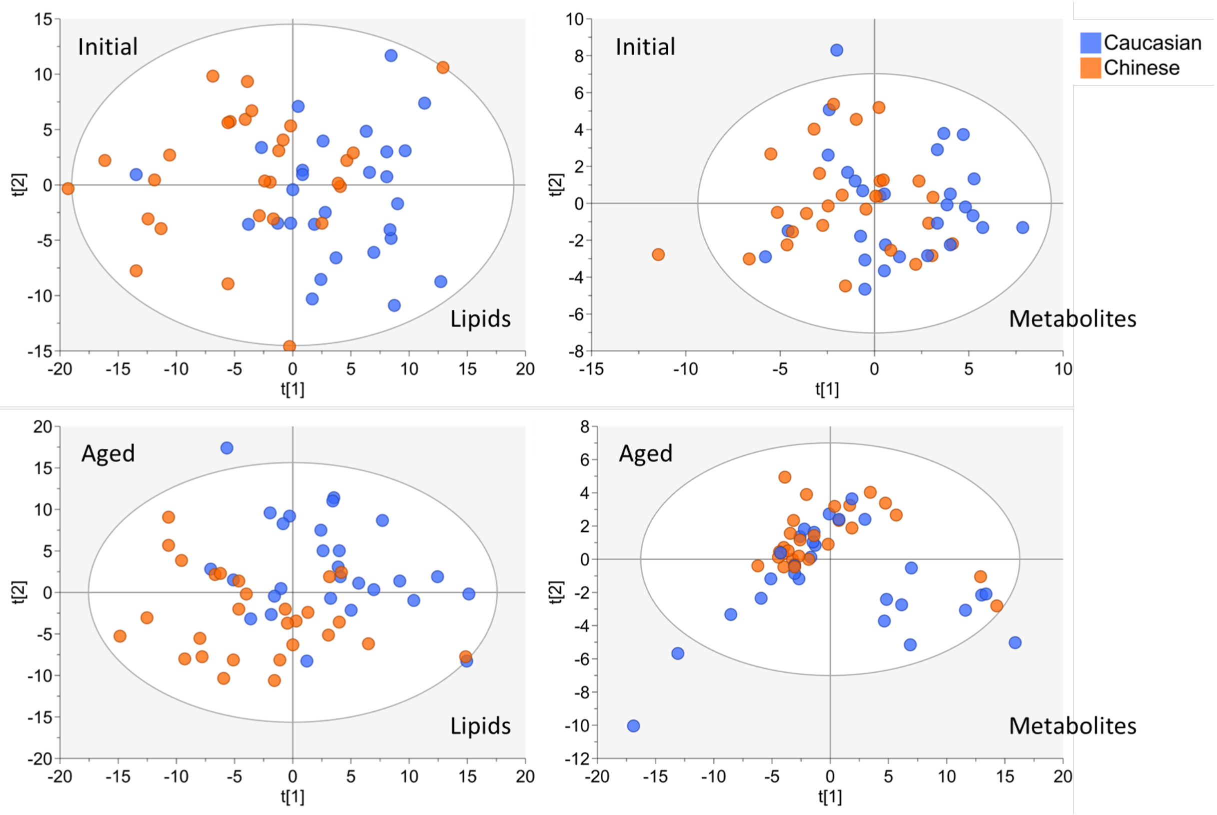

Figure S1. Principal component analysis of both Initial and Aged lipid and metabolite datasets. For each model, participant symbols were colored according to ethnicity (Caucasian blue symbols, Chinese orange symbols).

Supplementary Tables:

Table S1. Gradient elution programs per study analysis.

<table>
<tr><th></th><th colspan="2">Lipids</th><th colspan="2">Metabolites</th></tr>
<tr><th>Time (min)</th><th>Initial</th><th>Aged</th><th>Initial</th><th>Aged</th></tr>
<tr><td>0</td><td rowspan="2">5–30% B (0.0–2.0 min)</td><td rowspan="2">10–45% B (0–2.7 min)</td><td>3–3% A (0.0–1.0 min)</td><td>3–3% A (0.0–1.0 min)</td></tr>
<tr><td>1</td><td rowspan="11">3–30% A (1.0–12.0 min)</td><td rowspan="10">3–30% A (1.0–11.5 min)</td></tr>
<tr><td>2</td><td>30–48% B (2.0–2.5 min)</td><td>45–53% B (2.7–2.8 min)</td></tr>
<tr><td>3</td><td rowspan="8">48–82% B (2.5–11.0 min)</td><td rowspan="6">53–65% B (2.8–9 min)</td></tr>
<tr><td>4</td></tr>
<tr><td>5</td></tr>
<tr><td>6</td></tr>
<tr><td>7</td></tr>
<tr><td>8</td></tr>
<tr><td>9</td><td>65–89% B (9–9.1 min)</td></tr>
<tr><td>10</td><td>89–92% B (9.1–11 min)</td></tr>
<tr><td>11</td><td>82–99% B (11.0–11.5 min)</td><td>92–100% B was held for 0.8 min</td><td rowspan="2">30–90% A (11.5– 13.5 min)</td></tr>
<tr><td>12</td><td rowspan="3">99% B was held for 3.5 min</td><td rowspan="3">10% B for 3 min</td><td rowspan="3">30–90% A (12.0– 14.5 min)</td></tr>
<tr><td>13</td><td>90% A was held for 0.5 min</td></tr>
<tr><td>14</td><td rowspan="2">3% A for 2 min</td></tr>
<tr><td>15</td><td rowspan="3">15% B for 3 min</td><td rowspan="6"></td><td rowspan="3">90% A was held for 3.5 min</td></tr>
<tr><td>16</td><td rowspan="5"></td></tr>
<tr><td>17</td></tr>
<tr><td>18</td><td rowspan="3"></td><td rowspan="3">3% A for 3 min</td></tr>
<tr><td>19</td></tr>
<tr><td>20</td></tr>
</table>

HPLC gradient elution programs for both Initial and Aged Lipidomics and HILIC Metabolomics untargeted methodologies.

Table S2A: Lipidomic features of interest.

Initial Lipids		Aged Lipids		Initial Lipids		Aged Lipids		Initial Lipids		Aged Lipids	
m/z	rt	*m/z*	rt	*m/z*	rt	*m/z*	rt	*m/z*	rt	*m/z*	rt
496.34	1.62	496.34	2.67	744.55	6.75	744.55	7.54	786.60	6.57	786.60	7.30
520.34	1.30	520.34	2.49	744.59	6.32	744.59	6.92	787.67	7.89	787.67	8.97
524.37	2.54	524.37	3.53	744.59	6.77	744.59	7.50	788.56	5.64	788.55	6.69
544.34	1.23	544.34	2.45	746.57	6.05	746.57	6.57	788.62	7.17	788.62	8.05
610.54	7.18	610.54	8.54	746.61	6.89	746.60	7.62	790.57	5.80	790.57	6.48
612.56	7.79	612.56	9.43	748.53	5.98	748.53	6.72	790.69	9.96	790.69	10.67
634.54	6.64	634.54	7.87	748.59	6.60	748.58	7.19	792.55	5.20	792.55	7.24
636.56	7.23	636.56	8.69	750.54	6.26	750.54	7.04	792.59	6.02	792.59	6.62
638.57	7.83	638.57	9.58	754.53	5.66	754.54	6.15	792.59	5.67	792.59	6.77
666.62	11.45	666.62	11.17	754.54	5.03	754.54	5.52	794.57	5.61	794.57	6.18
675.54	4.85	675.54	5.22	756.55	5.35	756.55	5.85	794.60	6.19	794.60	6.92
688.60	10.71	688.60	10.94	759.64	7.15	759.64	7.86	794.60	6.76	794.60	7.66
689.56	5.22	689.56	5.56	760.58	6.40	760.59	7.03	796.58	6.05	796.58	6.72
690.62	11.16	690.62	11.07	762.60	7.10	762.60	7.89	796.62	6.92	796.62	7.84
697.52	4.85	697.52	5.22	764.52	5.69	764.52	6.30	799.67	7.58	799.67	8.39
700.53	6.39	700.52	7.04	764.56	5.57	764.56	6.15	801.68	8.27	801.68	9.56
701.56	4.95	701.56	5.35	764.68	9.91	764.68	10.64	802.53	5.27	802.53	5.79
705.59	5.84	705.59	6.23	766.54	4.94	766.54	6.58	804.55	5.36	804.55	5.88
706.54	5.63	706.54	6.01	766.57	6.02	766.57	6.25	806.57	5.25	806.57	6.48
714.62	10.89	714.62	10.98	766.57	5.65	766.57	6.68	807.63	7.21	807.63	8.05
716.52	6.01	716.52	6.57	768.55	5.40	768.55	5.89	808.58	6.60	808.58	7.31
717.59	5.92	717.59	6.36	768.55	6.61	768.55	7.43	808.58	5.77	808.58	6.38
718.54	6.59	718.54	7.26	768.59	6.17	768.59	6.82	809.65	7.90	809.65	7.34
718.57	6.73	718.57	7.36	770.57	5.63	770.57	6.19	809.65	6.55	809.65	7.24
723.54	4.95	723.54	5.35	770.60	6.37	770.60	7.02	810.60	7.17	810.60	6.74
724.53	6.22	724.53	6.94	770.61	6.93	770.60	7.79	810.60	6.44	810.60	6.95
725.56	5.61	725.56	5.95	772.58	6.20	772.58	6.68	811.66	6.59	811.66	6.54
727.57	5.11	727.57	6.23	772.62	7.09	772.62	7.94	811.67	7.18	811.67	8.05
728.56	7.13	728.56	8.07	773.65	7.54	773.65	8.42	812.62	6.76	812.62	7.60
729.59	5.69	729.59	6.14	774.54	6.04	774.54	6.84	813.68	7.85	813.68	9.18
730.54	5.16	730.54	5.58	774.60	6.77	774.60	7.51	814.63	7.24	814.63	8.23
731.61	6.37	731.61	6.83	776.56	6.74	776.56	7.71	815.70	9.13	815.70	10.11
732.55	5.69	732.55	6.17	778.54	4.86	778.54	5.83	818.60	5.96	818.60	6.71
733.62	6.62	733.62	7.17	780.55	5.86	780.55	6.39	818.72	10.47	818.72	10.78
734.57	6.37	734.57	6.88	780.55	5.27	780.55	5.79	820.58	5.84	820.58	6.54
740.52	5.89	740.52	6.49	782.57	6.38	782.57	7.03	820.62	6.23	820.62	7.59
742.57	6.19	742.57	6.79	782.57	5.70	782.57	5.90	820.62	6.66	822.64	7.89
744.55	5.51	744.55	5.95	785.65	7.20	785.65	7.87	822.64	6.90	823.66	9.56

Table S2A: continued: Lipidomic features of interest.

Initial Lipids		Aged Lipids		Initial Lipids		Aged Lipids	
m/z	rt	*m/z*	rt	*m/z*	rt	*m/z*	rt
823.67	8.27	823.68	10.77	881.75	11.77	881.76	11.14
823.68	10.46	828.55	5.82	883.77	12.14	883.77	11.28
828.55	5.58	829.72	10.25	884.77	12.14	884.77	11.28
829.72	8.84	830.57	5.67	886.79	11.23	886.79	10.96
830.57	5.07	832.58	7.20	888.80	11.62	888.80	11.07
832.58	5.60	832.74	10.83	890.82	10.94	890.81	11.20
832.74	10.72	834.60	7.61	892.74	10.13	892.74	10.70
834.60	5.84	834.60	6.61	892.83	12.25	892.83	11.34
834.60	6.23	834.60	7.01	894.75	10.23	894.75	10.77
835.67	7.86	835.67	8.95	898.78	11.04	898.79	10.93
836.62	6.73	836.62	7.28	899.71	10.23	899.71	10.76
837.68	8.64	837.68	10.11	901.73	10.63	901.73	10.83
838.63	6.98	838.63	7.95	901.73	10.86	902.73	10.84
842.72	10.14	842.72	10.72	902.82	11.81	902.82	11.14
844.74	10.55	844.74	10.79	903.74	11.05	903.74	10.93
846.75	10.96	846.76	10.89	904.59	5.68	904.59	6.23
848.77	11.37	848.77	11.00	904.74	11.07	904.74	10.94
849.69	10.54	849.69	10.79	904.83	12.13	904.83	11.28
850.67	7.63	850.67	8.95	905.75	11.47	905.76	11.03
851.71	10.94	851.71	10.89	907.77	11.83	907.77	11.15
853.72	11.35	853.72	11.00	909.79	12.13	909.79	11.28
854.57	5.61	854.57	6.22	916.83	10.97	916.83	11.22
855.74	11.79	855.74	10.91	924.80	11.25	924.80	10.97
856.58	6.24	856.58	7.00	925.73	10.64	925.72	10.82
858.75	10.79	858.75	10.85	926.82	11.71	926.82	11.09
860.77	11.19	860.77	10.95	929.76	11.27	929.76	10.97
862.79	11.60	862.78	11.07	930.84	11.82	930.85	11.28
866.72	9.98	866.72	10.68	932.86	11.37	932.86	11.44
868.74	10.26	868.74	10.74	944.77	11.45	944.77	10.76
870.75	10.66	870.76	10.82	944.86	11.40	944.86	11.03
872.77	11.02	872.77	10.91	946.79	11.91	946.78	10.83
874.78	11.40	874.78	11.44	948.80	10.65	948.80	10.93
875.71	10.62	875.71	10.82	950.81	10.85	950.81	10.96
877.72	11.02	877.72	10.91	960.89	11.79	960.89	11.61
878.70	8.33	878.70	10.07	968.77	11.16	968.77	10.74
878.82	12.14	878.81	11.27	970.79	10.49	970.78	10.80
879.74	11.40	879.74	11.02				
880.59	5.72	880.59	6.28				

Table 2B: Metabolomic features of interest.

Initial Metabolites		Aged Metabolites		Initial Metabolites		Aged Metabolites	
m/*z*	rt	*m*/*z*	rt	*m*/*z*	rt	*m*/*z*	rt
61.04	7.63	61.04	2.02	147.1137	15.85	147.1123	9.64
70.07	11.21	70.06	8.20	148.0426	10.74	148.0439	7.82
72.08	11.33	72.08	8.35	150.0591	10.99	150.058	8.09
76.04	13.59	76.04	10.33	152.0385	12.88	152.0365	9.99
84.05	13.56	84.04	10.51	153.0667	3.75	153.0654	1.16
86.10	10.60	86.10	7.74	156.0777	15.73	156.0764	9.70
90.06	12.90	90.05	9.72	157.0616	12.66	157.05991	10.28
102.06	13.10	102.05	10.01	159.0773	14.09	159.07581	11.23
104.03	13.92	104.04	10.64	160.1342	9.39	160.1335	5.21
104.07	10.83	104.07	7.65	160.8412	12.94	160.8419	8.81
106.05	13.94	106.05	10.86	162.1133	10.97	162.11301	6.68
111.01	13.58	111.01	10.30	164.0707	9.95	164.0722	7.19
116.07	11.20	116.07	8.20	164.8352	12.94	164.83611	6.90
118.05	13.01	118.05	9.77	166.0733	9.23	166.07179	4.90
118.09	11.32	118.09	8.35	166.0871	10.24	166.08611	7.49
118.09	9.60	118.09	6.50	167.0203	11.25	167.0213	9.97
120.07	13.10	120.07	10.02	168.0235	11.25	168.0248	10.04
123.06	2.56	123.05	1.01	169.0592	13.54	169.0578	10.51
124.01	13.31	124.01	8.72	170.0932	15.69	170.092	9.01
126.00	13.32	126.00	8.74	173.1294	11.37	173.127	8.40
126.02	13.33	126.02	9.01	175.1199	16.16	175.1185	9.53
127.05	13.47	127.05	10.31	176.1038	14.08	176.1024	11.23
128.03	13.47	128.03	10.31	176.1291	9.33	176.12759	6.28
129.07	12.72	129.07	9.73	182.082	11.86	182.0809	9.01
130.05	13.56	130.05	10.51	184.0946	11.87	184.0939	7.83
131.05	13.86	131.05	10.45	185.0332	13.54	185.0316	10.50
131.08	14.02	131.08	9.03	188.129	10.30	188.1275	6.00
132.08	12.61	132.08	9.30	189.1607	15.24	189.1591	8.56
132.10	10.28	132.10	7.57	191.019	12.55	191.0199	8.81
132.10	10.60	132.10	7.73	195.8102	12.98	195.8109	5.94
133.06	13.87	133.06	10.66	200.0416	15.70	200.04041	9.73
133.10	15.93	133.10	10.09	203.0535	12.21	203.0519	7.78
137.05	8.87	137.05	4.34	203.1512	15.04	203.1498	8.01
138.06	8.04	138.05	7.12	204.124	9.03	204.1226	3.74
138.06	10.21	138.05	4.59	205.0981	10.88	205.09689	7.94
144.10	9.15	144.10	6.26	209.093	10.50	209.09129	7.66
145.06	13.47	145.06	10.32	214.0599	14.07	214.05811	11.22
146.08	11.22	146.08	7.68	229.1558	9.37	229.1539	6.11
147.08	13.56	147.08	10.51	248.1488	8.51	248.1483	6.02
